# Death of Prof. E. K. Sanburn

**Published:** 1862-06

**Authors:** 


### EDITORIAL DEPARTMENT.

### DEATH OF PROF. E. K. SANBURN,

We are pained and saddened at the recent intelligence of the death of our former friend and class-mate, Dr. Eben K. Sanborn, Surgeon of the 31st Massachusetts Regiment, and Professor of Surgery in the Berkshire Medical College. He died at Ship Island from disease, thought to have been induced by over exertion. With the deepest sorrow we record the death of one so gifted, and who in his official relations, with the community and the profession, has been so highly esteemed. His loss will be severely felt, as few men of his age, had attained so high a position.

He was educated under the instruction of Dr. Kimball, of Lowell, one of the first surgeons in the country, and graduated in medicine at the Berkshire Medical College, in the class of 1847. After a time of study and observation in several European Hospitals, he returned to this country, and held the Professorship of Surgery in both the Berkshire and Vermont Medical Schools, where he had earned a well deserved reputation in his department of surgery.

At the commencement of the war he left a lucrative practice and the duties of his professorship and went with a Vermont regiment to Fortress Monroe, and as Post Surgeon at Newport News, was very efficient. His health was seriously impaired while there; but after a brief sojourn in Rutland, he entered again into the service of his country. He was one of the most enthusiastic of his profession, of highly inventive genius, possessed of rare qualities for the various emergencies of military surgery.

He died April 3d, and his remains were shipped in the Black Prince for Boston, but on the 9th, on account of some imperfection in embalming, it became necessary to consign the body to the deep, which was done with appropriate funeral solemnities. Thus ends the mortal life of one, of the many of our profession, who have gone at the call of duty and country, if not to face the cannon's mouth, to brave not only its dangers, but to war with another and more deadly foe; to fight hand to hand with death; not death, as it comes kindly and gently to relieve the worn sufferer in the flower scented chamber of opulence, after long years of gradual decline; not death as it comes with loved ones near to wipe the cold brow and smooth a dying pillow, but grim, ghastly and unwelcome; as it comes in battle, in camp, in plague; as it comes in all the untold and inconceivable horrors of war.—Our country requires the sacrifice and God will remember the true and faithful; yet we mourn that one so young and brave, should die.

